# Editorial for the Special Issue: Functional Polymer Composites

**DOI:** 10.3390/polym13060909

**Published:** 2021-03-16

**Authors:** Phuong Nguyen-Tri, Tuan Anh Nguyen

**Affiliations:** 1 Département de Chimie, Biochimie et Physique, Université du Québec à Trois-Rivières (UQTR), Trois-Rivières, QC G8Z 4M3, Canada; 2 Laboratory of Advanced Materials for Energy and Environment (Nguyen-Tri Lab.), Université du Québec à Trois-Rivières (UQTR), 3351 Boulevard des Forges, Trois-Rivières, QC G8Z 4M3, Canada; 3 Institute for Tropical Technology, Vietnam Academy of Science and Technology, Hanoi 122100, Vietnam; ntanh2007@gmail.com

Functional polymer composites are now making significant strides in synthesis, design, preparation, processing, and promising applications. Today, polymer composites are becoming lighter, cheaper, more durable, and more versatile. At present, great progress has been made in the design, preparation, and characterization of polymer composite materials, making them smarter and versatile. By creating new properties using suitable fillers and matrix, functional polymer composites can meet the most difficult standards of users, especially in high-tech industries. Functional polymer composites are crucial to the development of new cutting-edge technologies, especially in the fourth industrial revolution. Advances in functional polymer composites will promote human welfare by creating new solutions and materials to overcome global energy and environmental crises.

Advanced polymer composites reinforced by high-performance carbon fibers and nanofillers are popular in the automotive and aerospace industries thanks to their significant advantages, such as high specific strength to weight ratio and non-corrosion properties. In addition to the improvement of the mechanical performance, polymer composites today are designed to provide new functions dealing with antibacterial, electrical conducting, superhydrophobic, self-cleaning, self-healing, self-healing, self-actuating, biocompatibles, super-hard, solar reflective, heath monitoring and diagnosis, and high energy storage and conversion, for desired end-use applications. On the other hand, composite materials can contribute to reducing environmental issues by providing renewable energy technologies in conjunction with multifunctional, lightweight energy storage systems with high performance and noncorrosive properties. They are also used to prepare a new generation of batteries and directly contribute to H_2_ production or CO_2_ reduction in fuels and chemicals.

In this Special Issue, we have collected fifteen interesting articles from leading international researchers working in the multifunctional composites and polymer composites coming from 16 different countries including USA, Germany, Denmark, Canada, United of Kingdom (UK), Japan, France, Luxembourg, Korea, Australia, Malaysia, Poland, Vietnam, China and Egypt and Ukraine.

Authors showed their latest research in the field of smart and functional composites. Nguyen-Tri et al. [1] reviewed various advanced nanoscale techniques for the characterization of polymers and composites. They showed the recent development of atomic force microscopy (AFM) alone or combined with other techniques like infrared AFM (AFM-IR) for providing both morphological images at the nanoscale and chemical information. The emerging applications of these techniques in polymers, composites and crystallization of polymers are also presented and discussed. Traditional material composites with enhanced mechanical properties by adding carbon fiber are also reported. The latest advanced technology in cutting and drilling by using a fiber laser is also reported and discussed in the article of Ahmad Sobri et al. [2]. They also reported another interesting article on the effect of the machining input condition, especially the presence of entry and exit holes on the delamination of the composite layers [3]. Some mathematical models, designed for the calculation of resistance to the impact of fabric/polymer, have been presented and discussed by Horiashchenko et al. [4]. Vu et al. computed a Density Functional Theory (DTF )prediction model to calculate the electron density and bond distance of a conjugated polymer with different substitution groups and side-chain functional groups (H, Br, OH, OCH_3_ and OC_2_H_5_) [5].

The relationship between the experimental and theorical results of a synthetic rubber, Ethylene propylene diene monomer (EPDM), reinforced by nano size boron nitride is reported by Tariq Nazir et al. [6]. They indicated that experimental results could improve carbonized tracking lifetime with the addition of nano-BN (boron nitride) contents. Furthermore, surface temperature was reduced in the critical contamination flow path. The third harmonic component in the leakage current declined with the increase in the nano-BN contents. The improvement of thermal properties of Polybutylene succinate (PBS) composites reinforced by different rates of silicon carbide (SiC) by extrusion has been reported by Chernet Lule et al. [7]. Another interesting research paper focusing on the design of Fiber Reinforced Polymers (FRPs) in popular building is reported by a research group from the University of Stuttgart, Stuttgart, Germany [8]. They developed a parametric design tool for global design and fiber layout generation [9].

Kizhakidathazhath et al. [10] designed new polymer dispersed liquid crystals (PDLCs) by using mainly acrylate monomer (A3DA) with other additives, reducing the driving voltage without diminishing other revolutionary features. They also showed some exception properties of these materials that can be used for high-driving voltage materials. New composites based on organic aluminum hypophosphite (ALCPA) and its hybrid (CNALCPA) with graphitic carbon nitride (g-C_3_N_4_) were successfully synthesized and applied as halogen-free flame retardants in polyamide 6 (PA6) [11]. Finally, a new conductive, weft-stretchable fabric has been designed and prepared by Wang et al. [12]. The morphology, structure and properties of this new material have been also reported and discussed. The preparation and characterization of acrylic/ZnO is also reported [13]. An interesting antibacterial polymer made of thermoplastic Starch/Zinc Oxide Nanocomposite Films is also prepared and discussed [14]. New butadiene rubber composites reinforced by leather waste fiber are also reported in a recent article [15]. The possibility of using commercial printing equipment for recording, storing, and reproducing information on the shape memory polymer films is also evaluated [16]. Finally, Ziying Wang et al. [17] reviewed various natural functional biopolymers and their emerging applications in the electronic skins and flexible strain sensors 

## Data Availability

Not applicable.
